# Pituitary-Related Adverse Events and Onset Patterns Caused by Immune Checkpoint Inhibitors: Analysis Using the Japanese Adverse Drug Event Report Database

**DOI:** 10.3390/medicina59111963

**Published:** 2023-11-07

**Authors:** Hiroki Asano, Yoshihiro Noguchi, Michio Kimura, Eiseki Usami, Tomoaki Yoshimura

**Affiliations:** 1 Department of Pharmacy, Ogaki Municipal Hospital, 4-86 Minaminokawa-cho, Ogaki-shi 503-8502, Gifu, Japan; 2 Laboratory of Clinical Pharmacy, Gifu Pharmaceutical University, 1-25-4 Daigakunishi, Gifu-shi 501-1196, Gifu, Japan; yoshimurat@gifu-pu.ac.jp

**Keywords:** disproportionality analysis, pituitary-related adverse events, onset patterns, immune checkpoint, spontaneous reporting system, Japanese Adverse Drug Event Report (JADER) database

## Abstract

*Background and Objectives*: One type of immune-related adverse event caused by immune checkpoint inhibitors (ICIs) is pituitary-related adverse events. The management of pituitary-related adverse events is important because they can be fatal if not treated promptly. Therefore, this study was conducted to investigate the onset of pituitary-related adverse events using the Japanese Adverse Drug Report (JADER) database. *Materials and Methods*: Cases registered in the JADER database from 2004 to 2019 were used. The target drugs were ipilimumab, nivolumab, pembrolizumab, avelumab, atezolizumab, and durvalumab, and the target adverse events were the high-level terms “Anterior pituitary hypofunction,” “Anterior pituitary hyperfunction,” “Posterior pituitary disorder,” and “Pituitary neoplasm” in the Medical Dictionary for Regulatory Activities, Japanese version (MedDRA/J). The information component (IC) was used for signal detection and IC delta (ICΔ) was used for women-related signals. Onset timing and patterns were analyzed using the Weibull distribution. *Results*: Signals were detected with ipilimumab, nivolumab, pembrolizumab, and atezolizumab in “Anterior pituitary hypofunction,” with ICs and 95% credible intervals (95%CrI) of 5.53 (5.30–5.69), 4.96 (4.79–5.08), 4.04 (3.76–4.25), and 2.40 (1.53–3.00). Significant signals were detected in women, except for atezolizumab. Additionally, the time of onset was classified as the wear-out failure type. Inverse signals were detected with ipilimumab and nivolumab in “Posterior pituitary disorder,” with ICs (95%CrI) of −1.24 (−2.80–−0.26), and −0.89 (−1.64–−0.37). *Conclusions*: Anterior pituitary hypofunction is likely to occur with the long-term administration of ipilimumab, nivolumab, and pembrolizumab. Further investigation is needed to determine the differences in the tendencies to detect signals in the anterior and posterior pituitaries between ipilimumab and nivolumab.

## 1. Introduction

Immune checkpoint inhibitors (ICIs) enhance T-cell-mediated anti-tumor responses by targeting immune regulatory molecules such as cytotoxic T-lymphocyte-associated protein 4 (CTLA-4) and programmed death-1 (PD-1) and its ligand, PD-L1, and are effective against a variety of advanced malignancies [1]. Immune checkpoints are involved in maintaining the homeostasis of immune responses and are deeply involved in peripheral tolerance and the autoimmune diseases caused by its breakdown [2]. Immune dysfunction is associated with autoimmune diseases (ADs) and cancer. Preexisting autoimmune disease (PAD) is found in about 10% of cancer patients [3,4]. The etiology of irAE has yet to be fully understood. However, several mechanisms have been proposed. Autoreactive T cells may contribute to irAE due to their antigen sharing with tumor cells, increased inflammatory cytokine production through activation of the Th1 and Th17 pathways (e.g., colitis), antibody-dependent cytotoxicity induced by ectopic expression of CTLA-4 (e.g., pituitary inflammation), and modulation of antibody production of B cells by tumor-reactive T cells [5,6,7,8]. Thus, ICIs can cause immune-related adverse events (irAEs) [9], in which autoimmune mechanisms are implicated in the endocrine organs, lungs, gastrointestinal tract, liver, kidney, skin, nerves, muscles, and other organs throughout the body [10,11].

Endocrine disorders are one of the most common irAEs, and one endocrine irAE is hypophysitis. Hypophysitis is the second most common endocrine disease after thyroiditis [12,13] and requires proper management, as it can lead to adrenal crisis and death if not treated promptly [14,15]. The frequency of hypophysitis due to ICIs varies by drug class, ranging from 4% to 10% for anti-CTLA-4 antibodies and 0.3% to 1% for anti-PD-1 antibodies [16,17,18]. In addition, hypophysitis due to anti-CTLA-4 antibodies is said to be more common in men [16]. As anterior pituitary hormone abnormalities are frequently observed and diabetes insipidus due to posterior pituitary hormone dysfunction is rare, it is believed that the main body of pituitary inflammation is the anterior rather than the posterior pituitary. Thus, there is insufficient information on posterior pituitary disorders, and clarifying the effects of ICIs on the anterior and posterior pituitaries and sex differences may provide useful information for managing their side effects.

Recently, it has been reported that patients receiving ICIs who develop irAEs including pituitary-related adverse events have a significantly longer overall survival rate than those who do not [19,20,21], suggesting that side effect management may be a predictor of treatment efficacy. Therefore, the management of side effects is becoming increasingly important. Spontaneous reporting systems are attracting attention as one of the tools available for pharmacovigilance. The major spontaneous reporting systems include the Food and Drug Administration Adverse Event Report System (FAERS) in the United States and the Japanese Adverse Drug Event Report (JADER) database published by the Pharmaceuticals and Medical Devices Agency (PMDA) in Japan. Based on these findings, the clinical characteristics of patients with ICI-induced colitis [22] and the timing of onset of ICI-induced autoimmune disease have been reported [23]. Safety signals detected by spontaneous reporting systems are known to be sensitive to a small number of reports, allowing early detection of unknown adverse events. Since sufficient analysis cannot be performed if the number of cases is limited to those conducted only at our hospital, we decided to utilize a data mining method [24] based on the JADER. The aim of this study was to characterize the onset timing and pattern of pituitary disorders after the use of ICIs by distinguishing between anterior and posterior pituitary disorders and evaluating the signals in the anterior and posterior pituitaries.

## 2. Materials and Methods

### 2.1. Data Source

The data source for this analysis was JADER, provided by the PMDA, from 2004 Q1 to 2019 Q4. JADER consists of four tables with four comma-separated values (csv): case list (demo.csv), drug information (drug.csv), adverse event information (reac.csv), and primary disease (hist.csv). Demo.csv contains basic patient information, such as sex and age; drug.csv contains the generic name of the drug, route of administration, and start and end dates of administration; reac.csv contains the name of the adverse event, outcome, and date of adverse event occurrence; and hist.csv contains information on the underlying disease. Although the drug table classifies the involvement of drugs in adverse drug reactions into “suspect drug”, “concomitant drug”, and “interaction”, only “suspect drug” was included in this study.

### 2.2. Target Adverse Events and Target Drugs

Preferred terms (PTs) from the Japanese Medical Dictionary for Regulatory Activities, Japanese version (MedDRA/J), were used to identify adverse events. Standardized MedDRA Queries (SMQs) are available for grouping PTs; however, because the adverse events under investigation were not found in SMQs, they were evaluated using high-level terms (HLTs) [24]. PTs included in the HLTs for “Anterior pituitary hypofunction”, “Anterior pituitary hyperfunction”, “Posterior pituitary disorder”, and “Pituitary neoplasm” were included as pituitary-related adverse events. The ICIs used in this analysis were the anti-CTLA-4 antibody ipilimumab (Ipi), anti-PD-1 antibodies nivolumab (Nivo) and pembrolizumab (Pembro), and anti-PD-L1 antibodies atezolizumab (Atezo), avelumab (Avelu), and durvalumab (Durva). The spontaneous reporting system includes data on dosage but does not register dosage times. Since the spontaneous reporting system only contains data on patients who have experienced adverse drug reactions, the registered doses are not necessarily representative of the patients who use the drug. Therefore, these data could not be considered in the analysis.

### 2.3. Subsection

#### 2.3.1. Disproportionality Analysis

A disproportionality analysis was performed using a Bayesian confidence propagation neural network (BCPNN). The BCPNN is a method used by the World Health Organization–Uppsala Monitoring Center (WHO-UMC), which uses the information component (IC) as a signal score. IC scores were calculated using a 2 × 2 contingency table (Table 1) and Equations (1) and (2). ICs were defined as signals if the lower limit of the 95% credible interval (95%CrI) (IC_025_) was >1 and inverse signals if the upper limit of the 95%CrI was <1 [25]. The JADER database used in this study registered 692,917 adverse events reports. This number of reports is the *N*_++_ (Table 1) used to calculate the IC.

(1) $$OE=\frac{O(\mathrm{Observed})}{E(\mathrm{Expected})}=\frac{N_{11}}{N_{+1}N_{1+}/N_{++}}$$

(2) $$\mathrm{IC}\approx log_{2}\frac{O+0.5}{E+0.5}$$

Sandberg L. et al. proposed a method to highlight associations with significant contrasts between subgroups and the rest of the database—corresponding covariates (if the subgroup of interest is children). The adjusted OE ratio for the rest of the database is calculated as the weighted average of the OE ratio for the other subgroups. This simplifies the OE ratio, where the observed and expected numbers are summed across subgroups [26]. In this study, sex was used to create subgroups to investigate sex differences. To investigate the impact of sex on pituitary-related adverse events, IC delta (IC_Δ_) [27] scores were calculated for drugs for which a signal was detected in the overall population. IC_Δ_ scores were calculated using a 4 × 2 contingency table (Table 2) and Equations (3)–(6). IC_Δ_ was defined as a signal if the lower limit of the 95%CrI (IC_Δ025_) was >0. However, signal detection in women also met the following criteria: the number of reported targeted irAEs caused by targeted drugs in women (*N*_women11_) > 2 and IC_025_ for women (IC_women025_) > 0.

(3) $${\mathrm{OE}}_{\mathrm{women}}=\frac{O_{\mathrm{women}}\left(Observed\right)}{E_{\mathrm{women}}\left(Expected\right)}=\frac{N_{women11}}{N_{women+1}N_{women1+}/\left(N_{women+1}+N_{women+0}\right)}$$

(4) $${\mathrm{OE}}_{\mathrm{men}}=\frac{O_{\mathrm{men}}\left(Observed\right)}{E_{\mathrm{men}}\left(Expected\right)}=\frac{N_{men11}}{N_{men+1}N_{men1+}/\left(N_{men+1}+N_{men+0}\right)}$$

(5) $${OE}_{\Delta }=\frac{{OE}_{women}}{{OE}_{men}}=\frac{O_{women}}{E^{*}}$$

(6) $${\mathrm{IC}}_{\Delta }\approx log_{2}\frac{O_{\mathrm{women}}+0.5}{E^{*}+0.5}$$

#### 2.3.2. Weibull Analysis

The number of days until the onset of adverse events in the pituitary gland with each ICI was evaluated by estimating the scaling parameter α value and shape parameter β value using the Weibull distribution [28] (Equation (7)).

(7) $$\lambda (T)=\frac{\beta }{\alpha }{\left(\frac{T-\gamma }{\alpha }\right)}^{\beta -1}$$

**Figure 1:**
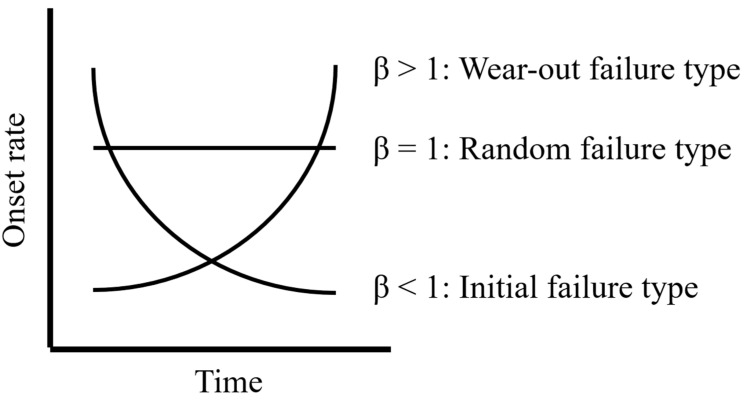
Shape parameters (β) and Weibull distribution.

The shape parameter β indicates the onset pattern of adverse events. Thus, when β > 1, the pattern is a wear-out failure type in which the onset rate increases with time; when β is 1, the pattern is a random failure type in which the onset rate remains constant regardless of time; and when β < 1, the pattern is an early failure type in which the onset rate decreases with time (Figure 1).

## 3. Results

### 3.1. Signal Score for Pituitary-Related Adverse Events for Each ICI

The ICs (95%CrI) for “Anterior pituitary hypofunction” were as follows: Ipi: 5.53 (5.30–5.69), Nivo: 4.96 (4.79–5.08), Pembro: 4.04 (3.76–4.25), and Atezo: 2.40 (1.53–3.00). Signals were detected in Ipi, Nivo, Pembro, and Atezo. The IC (95% CrI) for “Anterior pituitary hyperfunction” was as follows, with no signal detected: Nivo: −0.94 (−4.72–0.75). The ICs (95% CI) for “Posterior pituitary disorder” were as follows, with an inverse signal detected for Ipi and Nivo: Ipi: −1.24 (−2.80–−0.26), Nivo: −0.89 (−1.64–−0.37), Pembro: 0.16 (−0.49–0.63); and Atezo: 0.55 (−0.48–1.24). The ICs (95% CI) for “Pituitary neoplasm” were as follows, with no signal detected: Ipi: 0.62 (−3.17–2.30) and Nivo: −0.37 (−4.15–1.32) (Table 3).

“Anterior pituitary hypofunction” detected several signals; therefore, we also investigated the sex differences in the signal scores for the agents detected. Signals in women and men related to “Anterior pituitary hypofunction” were detected in all ICIs. The IC_Δ_ (95% CrI) for “Anterior pituitary hypofunction” was as follows: Ipi: 1.24 (0.85–1.52), Nivo: 1.09 (0.77–1.33), Pembro: 0.89 (0.26–1.34), and Atezo: 1.30 (−0.26–2.28). Among the four drugs studied, significant signals were detected in women, except for Atezo (Table 4). That is, women, but not men, showed a stronger association.

### 3.2. Analysis of the Onset Pattern of Pituitary-Related Adverse Events

The Weibull distribution was used to analyze the onset patterns in Ipi, Nivo, Pembro, and Atezo, for which signals were detected in anterior pituitary hypofunction. The shape parameter β (95% CI) was as follows: Ipi: 1.54 (1.36–1.73), Nivo: 1.50 (1.34–1.67), Pembro: 1.87 (1.54–2.22), and Atezo: 2.29 (0.92–4.49) (Table 5).

## 4. Discussion

The onset of pituitary-related adverse events associated with anti-CTLA-4 antibodies has been reported after approximately 10 weeks [16]; that associated with anti-PD-1 and anti-PD-L1 antibodies develops after several months to 1 year [29]; and combined clinical trial data for Nivo indicate onset after approximately 12 weeks [30]. Although there has been a paper [31] on the onset of irAE after ICIs using JADER, this is the first study to distinguish between disorders in the anterior and posterior pituitaries. This study evaluated the anterior and posterior pituitaries of patients with pituitary disorders treated with ICIs to characterize the onset timing and pattern of irAEs. Among the targeted ICIs, signals of “Anterior pituitary hypofunction” were detected in the Ipi, Nivo, Pembro, and Atezo groups (Table 3). Significant signals were detected in women, except for Atezo (Table 4). 

Some reports suggest that sex differences may not need to be considered for irAE management [32,33], while others suggest that tissue and organ toxicity of ICI is sex-specific [34,35]. Yang F et al. analyzed gene expression levels of ICIs in various tissues and organs of men and women with the GTEx portal (https://gtexportal.org/home/gene/ (accessed on 27 September 2023)). Gene expression levels were normalized using log10 (transcripts per million (TMP) + 1), and the pituitary had a median TPM value: men = 4.66 and women = 5.02. The expression levels of genes encoding immune checkpoints may indicate the response to ICIs in the corresponding tissues. Inhibition of its expression may cause dysfunction in these tissues, which may lead to organ-specific irAEs. Based on this, irAEs in the pituitary gland may be more likely to occur in women; a similar trend of signal detection was also observed in this study. A study by Unger JM et al. showed that women are at significantly higher risk of serious symptomatic AEs in multiple therapeutic areas, including patients receiving targeted ICI therapy [36]. However, there are also reports that the incidence rate is higher in men, so sufficient caution is required in interpreting the results [37].

The shape parameter β in the Weibull distribution of hypopituitarism (Table 5) for Ipi, Nivo, and Atezo exceeded 1; for Atezo, the value was close to 1, although the onset pattern could not be determined. Based on these findings, “Anterior pituitary hypofunction” by Ipi, Nivo, Pembro, and Atezo was classified as an abrasion failure type, and the frequency of its occurrence increased over time. Based on the α value of the scale parameter, approximately 60% of patients who developed adverse events after 100–200 days of treatment developed “Anterior pituitary hypofunction.” Therefore, these results indicate that “Anterior pituitary hypofunction” should be noted even in the early stages of administration. However, no signal was detected in Avelu or Durva.

The indications for both drugs in Japan (as of March 2020) were “radically unresectable Merkel cell carcinoma” (Approved September 2017) and “unresectable or metastatic renal cell carcinoma” (Approved December 2019) for Avelu and “maintenance therapy after radical chemoradiation in unresectable locally advanced non-small cell lung cancer” (Approved July 2018) for Durva. Thus, both drugs are intended for use in less frequent diseases and were recently approved, and the number of cases necessary for analysis did not increase.

An inverse “Posterior pituitary disorder” signal was detected in the Ipi and Nivo groups (Table 3). An inverse signal indicates may work in an inhibitory manner [38]. Among the pituitary-related adverse events caused by ICIs, diabetes insipidus is rare, occurring only 0.7% of the time, and has been reported to result from panhypopituitarism, a selective injury to the posterior pituitary gland or hypothalamus [39]. The mechanism underlying the higher prevalence of ICI-induced adverse events in the anterior pituitary than in the posterior pituitary or hypothalamus remains unclear. 

At present, it can only be concluded that anterior hypopituitarism and posterior pituitary disorders have different trends in signal detection and that Ipi and Nivo have different effects on the anterior and posterior pituitaries. However, these results support the hypothesis that the anterior rather than the posterior pituitary is the main site of pituitary-related adverse events.

This study also analyzed the HLT signals for pituitary-related adverse events, including “Anterior pituitary hyperfunction” and “Pituitary neoplasm.” Only one case of “Anterior pituitary hyperfunction” was reported in the Nivo group, and no signals were detected. Hypothalamic hormones regulate the secretion of anterior pituitary hormones, and factors in the hypothalamus promote and inhibit anterior pituitary hormone secretion [40]. Because prolactin secretion is inhibited and regulated by dopaminergic neurons in the hypothalamic arch nucleus, it is increased in hypothalamic disorders and hyperprolactinemia. As there has been a report of hypothalamitis caused by Atezo [41], “Anterior pituitary hyperfunction” caused by ICIs is possible. In the case of “Pituitary neoplasm,” only one case has been reported for both Ipi and Nivo, and no signals were detected. Adverse events [42] that have occurred since Nivo marketing approval include “benign, malignant, and neoplasms of unknown details.” However, no reports have discussed ICIs or pituitary neoplasms. The accumulation and re-evaluation of information on “Anterior pituitary hyperfunction” and “Pituitary neoplasm” are needed.

This study has some limitations specific to spontaneous reporting systems [24]. As JADER is a database based on spontaneous reports, and the number of patients using the drug is unknown, the incidence rates cannot be calculated [24]. Additionally, it was impossible to completely exclude the following reporting biases [24]: underreporting, the possibility that clinically unrecognized cases may not be enrolled; the Weber effect (an increase in the number of reports during the launch period) [43,44]; and notoriety effects (the number of adverse events reported increase overall) [45]. Moreover, the target drug was the ICIs alone; the effects of other drugs used in combination with the ICI cannot be evaluated. Although ICIs are used to treat various cancer types, this study did not investigate which ones they were used to treat. This is because a study using a spontaneous reporting system would have made comparisons of reported numbers inappropriate since the number of patients using the drug is unknown. The greater the number of patients using it, the greater the number of adverse events reported, even if the risk of occurrence is low. If the number of reports per cancer type were listed in this paper, the results could be misinterpreted as indicating a higher risk for the cancer type with the highest number of reports. Finally, The JADER contained some missing data. Missing sex data affect the signal of sex differences. In addition, there were several missing dosing data and/or onset dates in the Weibull analysis. Therefore, these results should be interpreted with caution.

Using a spontaneous reporting system makes it possible to conduct clinical research at a lower cost than other clinical research. Early detection of unknown adverse events is also possible; however, the inability to calculate accurate incidence rates is a weakness of this study.

In the future, ICIs are expected to expand the treatment options for more malignant tumors, and more detailed information for the identification of adverse effects is essential for the proper use of ICIs.

## 5. Conclusions

In this study, the frequency of anterior pituitary hypofunction caused by some ICIs increased over time, indicating the need to monitor the early stages of ICI administration, which will help in the management of pituitary-related adverse events caused by ICIs. The results also suggest that the effects of ICIs on the anterior and posterior pituitaries differ, although further investigation in clinical practice is needed.

## Figures and Tables

**Table 1:** The 2 × 2 contingency table for signal detection.

		Target Adverse Event	Other Adverse Events	Total
	
**Target drug**		*N*_11_	*N*_10_	*N*_1+_
**Other drugs**		*N*_01_	*N*_00_	*N*_0+_
**Total**		*N*_+1_	*N*_+0_	*N*_++_

**Table 2:** The 4 × 2 contingency table for women vs. men.

		Target Adverse Event	Other Adverse Events	Total
	
**Women**	**Target drug**	*N*_women11_	*N*_women10_	*N*_women1+_
	**Other drugs**	*N*_women01_	*N*_women00_	*N*_women0+_
**Men**	**Target drug**	*N*_men11_	*N*_men10_	*N*_men1+_
	**Other drugs**	*N*_men 01_	*N*_men00_	*N*_men0+_

**Table 3:** The number of reports and signal scores for adverse events related to the pituitary gland for each ICI.

Class	Drug	Anterior Pituitary Hypofunction		Anterior Pituitary Hyperfunction		Posterior Pituitary Disorder		Pituitary Neoplasm	
		*N*_11_	IC (95%CrI)	*N*_11_	IC (95%CrI)	*N*_11_	IC (95%CrI)	*N*_11_	IC (95%CrI)
anti-CTLA-4 antibody	ipilimumab	213	5.53 (5.30 *–5.69)	0	NA	5	−1.24 (−2.80–−0.26 ^†^)	1	0.62 (−3.17–2.30)
anti-PD-1 antibody	nivolumab	400	4.96 (4.79 *–5.08)	1	−0.94 (−4.72–0.75)	20	−0.89 (−1.64–−0.37 ^†^)	1	−0.37 (−4.15–1.32)
	pembrolizumab	134	4.04 (3.76 *–4.25)	0	NA	26	0.16 (−0.49–0.63)	0	NA
anti-PD-L1 antibody	atezolizumab	15	2.40 (1.53 *–3.00)	0	NA	11	0.55 (−0.48–1.24)	0	NA
	avelumab	0	NA	0	NA	0	NA	0	NA
	durvalumab	3	0.69 (−1.38–1.90)	0	NA	0	NA	0	NA

*N*_11_: the number of reports (see Table 1), IC: information component, 95% CrI: 95% credible interval, NA: not available, *: signal, ^†^: inverse signal.

**Table 4:** Comparison of women’s and men’s signal scores for anterior pituitary hypofunction by ICI.

Class	Drug	Women		Men		Women Versus Men
		*N*_women11_	IC (95%CrI)	*N*_men11_	IC (95%CrI)	IC_Δ_ (95%CrI)
anti-CTLA-4 antibody	ipilimumab	73	5.77 (5.39 *–6.05)	137	5.01 (4.73 *–5.21)	1.24 (0.85 **–1.52)
anti-PD−1 antibody	nivolumab	105	5.35 (5.03 *–5.59)	292	4.50 (4.31 *–4.64)	1.09 (0.77 **–1.33)
	pembrolizumab	28	4.14 (3.51 *–4.58)	104	3.65 (3.32 *–3.88)	0.89 (0.26 **–1.34)
anti-PD-L1 antibody	atezolizumab	5	2.60 (1.04 *–3.58)	9	1.86 (0.72 *–2.62)	1.30 (−0.26–2.28)

*N*: the number of reports (see Table 2), IC: information component, 95% CrI: 95% credible interval, *: signal, **: significant signal for women.

**Table 5:** Weibull parameters for each immune checkpoint inhibitor.

Class	Drug	*N*_11_	α (95%CI)	β (95%CI)
anti-CTLA-4 antibody	ipilimumab	131	97.3 (86.3–109.4)	1.54 (1.36–1.73)
anti-PD-1 antibody	nivolumab	164	158.5 (141.9–176.6)	1.50 (1.34–1.67)
	pembrolizumab	63	201.6 (174.6–231.7)	1.87 (1.54–2.22)
anti-PD-L1 antibody	atezolizumab	5	115.7 (67.6–194.7)	2.29 (0.92–4.49)
	avelumab	0	NA	NA
	durvalumab	0	NA	NA

*N*_11_: the number of reports (see Table 1), α: scale parameter, β: shape parameter, 95% CI: 95% confidence interval, NA: not available.

## Data Availability

The authors do not own the data because the Japanese authority, PMDA, does not permit sharing the Japanese Adverse Drug Event Report database (JADER) directly. Data owned by PMDA can be accessed directly here: http://www.info.pmda.go.jp/fukusayoudb/CsvDownload.jsp (accessed on 27 September 2023) (only in Japanese).
